# Disaster preparedness and response among individuals or their immediate family members lacking legal status (IFLLS) in the United States: a national survey

**DOI:** 10.1186/s12889-025-21693-9

**Published:** 2025-02-10

**Authors:** Christine Crudo Blackburn, Matthew R. Boyce, Mayra Rico, Kirk Niekamp, Jason Moats

**Affiliations:** 1 Department of Health Policy and Management, School of Public Health, Texas A&M University, 212 Adriance Lab Road, College Station, College Station, TX 77843 USA; 2 USA Center for Rural Public Health Preparedness, Texas A&M University, College Station, TX USA

**Keywords:** Disasters, Immigrants, Natural hazard, Preparedness, Public health, Vulnerable populations

## Abstract

**Background:**

Approximately 11 million immigrants without legal status live in the United States and many of these individuals live in areas that are prone to natural hazards. While there is sufficient literature to suggest that these individuals are more vulnerable to the impacts of disasters and are less prepared for disasters, there is limited understanding of natural disaster risk perception, behavioral intentions, and trust in disaster assistance among individuals or their immediate family members lacking legal status (IFLLS) in the United States. To address this gap, this study sought to describe and compare risk perception, barriers to evacuation, and trust in disaster assistance between legal citizens and IFLLS.

**Methods:**

A cross-sectional, online survey was conducted from April 24 to June 3, 2024. Survey respondents were asked about their demographic and socioeconomic characteristics, their risk perceptions and attitudes regarding natural hazards, reasons for not complying with evacuation orders, and their trust in assistance offered by various entities during the response to natural hazards. Pearson chi-square tests and Cramér’s V were used to investigate the association between IFLLS and variables of interest.

**Results:**

2,989 individuals completed the survey and 284 reported that they identified as IFLLS. Statistically significant relationships existed between IFLLS and residing in an area that experiences natural hazards, having been previously impacted by a disaster, anticipating being impacted by a natural hazard within the next 12 months, various reasons for noncompliance with evacuation orders, and trust in assistance offered during disaster response. There were not statistically significant relationships between IFLLS and the belief that preparedness for natural hazards is important or concern about natural hazards becoming more severe or frequent in the future.

**Conclusions:**

These results provide the first nationally representative examination of disaster risk faced by IFLLS in the United States, as well as the barriers to evacuation. These findings highlight the importance of developing disaster preparedness and response plans that incorporate the needs of IFLLS, as these groups face higher risk. Preparedness and response policies must consider and address the unique barriers faced by IFLLS.

## Introduction

There are an estimated 11 million immigrants without legal status in the United States (US) and most of these individuals live in mixed-status households [1], defined as households in which not all members of the immediate family have legal status. Importantly, while immigrants without legal status reside in every state, three of the ten most disaster-prone states—California, Texas, and Florida—also have the largest populations of immigrants without legal status [2]. The concentration of these populations in disaster prone areas increases the importance and urgency of understanding disaster preparedness and response in this population for disaster and public health planners and responders.

When natural hazards (e.g., hurricanes) occur, immigrants without legal status also encounter more barriers to effective disaster preparedness, response, and recovery when compared to native-born Americans and those with legal status [3–5]. Language is a primary obstacle that immigrants—both with and without legal status—face regarding disaster preparedness and response because emergency warnings and emergency information are often only available in English [6–8]. Even when emergency information is available in Spanish, the most common non-English language spoken in the US, there are thousands of immigrants whose primary language is neither English nor Spanish who are often unable to understand, much less comply with emergency warnings and directions [7].

Individuals or those with immediate family members lacking legal status (IFLLS) may also face limitations on their ability to travel outside of their community, making it more difficult for them to evacuate when a disaster is approaching [3, 9]. These limitations can be due to an unfamiliarity with surrounding communities and states making them unsure of where they can safely evacuate to [5]. Other times, fear of governmental entities or law enforcement discovering a lack of legal status restricts not only their willingness to evacuate when a disaster is approaching, but also their willingness to seek assistance once a disaster occurs [9, 10]. This unwillingness can be exacerbated in areas that experience high immigration enforcement, such as border states, because trust in governmental entities among some demographic groups (e.g., Hispanics and Latinos) declines in these contexts [11].

Previous research suggests that some disparities in preparedness can be overcome through culturally and linguistically competent disaster communication [12–14]. Still, gaps remain in preparedness between immigrants and native-born Americans in the US [15, 16]. Additionally, despite an understanding that immigrants are frequently left out of planning and response efforts [6], immigrants without legal status continue to be disproportionately affected by disasters and receive little to no public assistance during recovery [4]. These disparities mean that disasters often exacerbate existing inequalities between those with and without legal status in the US [7].

To date, there are limited studies examining natural hazard risk perception, behavioral intentions, and trust in assistance among IFLLS in the US. The objectives of this study, then, are to describe and compare risk perception, barriers to evacuation, and trust in disaster assistance offered by various actors between legal citizens and IFLLS. The findings from this study provide the first nationally representative examination of the disaster risk faced by IFLLS in the US, as well as barriers to evacuating to safety as a disaster approaches.

## Methods

To examine disaster preparedness and response among IFLLS, an online survey using the Qualtrics XM survey software (Seattle, WA) queried about natural hazard risks, attitudes, behavioral intentions, trust, and demographics. For the purposes of this survey, natural hazards referred to a variety of potential incidents arising as acts of nature including hurricanes, tornadoes, floods, snowstorms, heatwaves, earthquakes, landslides, avalanches, and other similar events [16]. Respondents were asked to select their state of residence, which was later coded by researchers according to the regional definitions used by the US Census Bureau [17]. The survey contained one attention check to ensure data quality [18]. The survey question used to determine the IFLLS identification of respondents queried, “Do you or anyone in your immediate family lack legal status in the United States?” The question was worded in this manner because the research team felt it would reduce fear among those without legal status or in mixed status families (i.e., they would not have to claim their individual legal status). Additionally, the question was at the end of the survey, on a separate page, and made optional for participants.

The survey platform Lucid was used to recruit respondents. Lucid provides a large, online, opt-in panel and offers incentives to survey respondents. The survey ran from April 24 through June 3, 2024, and surveys were administered in both English and Spanish. To be eligible for participation, respondents were required to reside in the US and be 18 years of age or older. Respondent quotas were implemented for age, gender, race/ethnicity, and geographic region to ensure a diverse and nationally representative sample.

### Data analysis

Descriptive data analysis presented the absolute and relative frequencies of the variables of interest. Pearson chi-square tests were used to investigate the association between IFLLS and risk perception and attitudes regarding natural hazards, reasons reported for noncompliance with evacuation orders, and trust in the assistance offered by various entities during the response to natural hazards. To analyze the relationship between IFLLS and the variables of interest, Pearson’s chi-square tests were performed, and the level of correlation was calculated using Cramér’s V. The threshold for statistical significance was set at an alpha value of 0.05 (*p* < 0.05) for all analyses, and analyses were conducted in August 2024 using StataSE v.18 (College Station, TX).

### Ethics approval

The study was declared exempt by the Texas A&M University Institutional Review Board following the determination that participation posed no more than minimal risk to survey participants. The research team conducted the study in compliance with the Declaration of Helsinki.

## Results

Overall, 3,012 adults (18 years and older) initiated the survey, and 2,989 respondents were included in the analysis. A majority of the respondents with legal status (i.e., non-IFLLS) were female, 54 years of age or younger, identified as white, had at least some college (or less), were employed, had an annual household income of $49,999 or less, resided in a Southern state, and resided in a suburban setting (Table 1). The data closely mirrored national population benchmarks on gender, race/ethnicity, income, and education [19]. Of the 2,989 respondents, 284 reported that they or someone in their immediate family lacked legal immigration status in the US. A majority of the respondents identified as IFLLS were male, 34 years of age or younger, identified as white, had a high school diploma (or less), were employed, had an annual household income below $49,999 or less, resided in a Southern state, and resided in a suburban setting. For the study, we report that all findings with a 95% confidence interval (margin of error of +/- 1.0%).

**Table 1:** Reported characteristics of survey respondents (*n* = 2,989)

Variable	Legal citizen *n* = 2705 (%)	IFLLS *n* = 284 (%)
Gender		
*Male*	1288 (47.6)	151 (53.2)
*Female*	1417 (52.4)	133 (46.8)
Age		
*≤ 34 years*	727 (26.9)	172 (60.6)
*35–44 years*	460 (17.0)	63 (22.2)
*45–54 years*	456 (16.8)	23 (8.1)
*55–64 years*	482 (17.8)	14 (4.9)
*65–74 years*	404 (14.9)	8 (2.8)
*≥ 75 years*	176 (6.5)	4 (1.4)
Race		
*White*	1680 (62.1)	114 (40.1)
*Asian*	106 (3.9)	24 (8.4)
*Black or African American*	313 (11.6)	64 (22.5)
*Hispanic or Latino*	478 (17.7)	72 (25.3)
*Other*	49 (1.8)	7 (2.5)
*Two or more races*	79 (2.9)	3 (1.0)
Education (highest level)		
*High school diploma or less*	983 (36.3)	126 (44.4)
*Some college or technical certification/degree*	683 (25.2)	56 (19.7)
*Associate’s or Bachelor’s degree*	808 (29.9)	71 (25.0)
*Graduate degree (Master’s*,* Doctoral*,* or professional)*	231 (8.5)	31 (10.9)
Employment Status		
*Employed (full- or part-time)*	1340 (49.5)	209 (73.6)
*Unemployed*	475 (17.6)	41 (14.4)
*Retired*	678 (25.0)	16 (5.6)
*Other*	212 (7.8)	18 (6.3)
Household income (USD)		
*< $25*,*000*	812 (30.0)	111 (39.1)
*$25*,*000–$49*,*999*	705 (26.1)	56 (19.7)
*$50*,*000–$74*,*999*	488 (18.0)	51 (17.9)
*$75*,*000–$99*,*999*	329 (12.2)	27 (9.5)
*$100*,*000–$149*,*999*	232 (8.6)	19 (6.7)
*≥ $150*,*000*	139 (5.1)	20 (7.0)
US Region		
*Northeast*	475 (17.6)	54 (19.0)
*Midwest*	505 (18.7)	50 (17.6)
*West*	661 (24.4)	66 (23.2)
*South*	1064 (39.3)	114 (40.1)
Residential Area		
*Urban*	772 (28.5)	92 (32.4)
*Suburban*	1198 (44.3)	111 (39.1)
*Rural*	735 (27.2)	81 (28.5)

Of the 284 respondents who identified as IFLLS, 228 reported living in an area that experiences natural hazards, 215 reported being previously impacted by natural hazards, and 204 anticipated being impacted by a natural hazard within the next 12 months; if proportionately equal to the non-IFLLS population, these frequencies were expected to be 201, 165, and 155, respectfully. A statistically significant relationship existed between IFLLS and residing in an area that experiences natural hazards (Χ^2^ (1, *n* = 2,989) = 14.19, *p* < 0.000), having been previously impacted by natural hazards (Χ^2^ (1, *n* = 2,989) = 39.61, *p* < 0.000), and anticipating being impacted by a natural hazard within the next 12 months (Χ^2^ (1, *n* = 2,989) = 50.04, *p* < 0.000) (Table 2), meaning that IFLLS are more likely to live in area that experience disaster, have been impacted by a disaster, and anticipate being impacted by a disaster in the future compared to non-IFLLS respondents. Further, 209 of those who reported they were IFLLS reported having an emergency evacuation plan, 225 reported having an emergency kit in their household, 246 reported that they believed preparedness for natural hazards to be important, 225 reported concern about the frequency of natural hazards in the future, and 227 reported concern about the severity of natural hazards in the future; if proportionately equal to the non-IFLLS population, the expected frequencies for these values were 157, 170, and 248, 220, and 222, respectfully. There was a positive statistically significant relationship between non-IFLLS status and having a personal or family evacuation plan (Χ^2^ (1, *n* = 2,989) = 41.57, *p* < 0.000), and having an emergency kit in one’s home (Χ^2^ (1, *n* = 2,989) = 49.48, *p* < 0.000), meaning those who were IFLLS are more likely to have these things. There was not a statistically significant relationship between IFLLS and the belief that preparedness for natural hazards is important (Χ^2^ (1, *n* = 2,989) = 1.10, *p* = 0.295), concern about natural hazards becoming more severe in the future (Χ^2^ (1, *n* = 2,989) = 1.76, *p* = 0.184), or concern about natural hazards becoming more frequent in the future (Χ^2^ (1, *n* = 2,989) = 1.58, *p* = 0.208). Cramér’s V suggested weak, positive correlations between IFLLS and residence in a disaster-prone area (v = 0.07), being previously impacted by disaster (v = 0.11), anticipating being impacted by a natural hazard within the next 12 months, having an emergency evacuation plan, and having an emergency kit in one’s house; negligible correlations existed between IFLLS and believing that preparedness for natural hazards is important (v = -0.02), concern about the severity natural hazards in the future (v = 0.02), and concern about the frequency natural hazards in the future (v = 0.03).

**Table 2:** Comparison of risk perception and attitudes regarding natural hazards between those who identified as IFLLS and those who did not

Variable			IFLLS		Pearson X^2^	Cramér’s V
			No (%)	Yes (%)		
Risks	Residence in disaster prone area	Yes	1882 (70%)	228 (80%)	*14.19	0.07
		No	823 (30%)	56 (20%)		
	Previously impacted by disaster	Yes	1524 (56%)	215 (76%)	*39.61	0.11
		No	1181 (44%)	69 (24%)		
	Anticipates future impact by disaster	Yes	1187 (63%)	204 (86%)	*50.04	0.15
		No	689 (37%)	32 (14%)		
Attitudes	Has an emergency evacuation plan	Yes	1450 (54%)	209 (72%)	*41.57	0.12
		No	1255 (46%)	75 (28%)		
	Has an emergency kit	Yes	1561 (58%)	225 (79%)	*49.48	0.13
		No	1144 (42%)	59 (21%)		
	Believes preparedness is important	Yes	2427 (98%)	246 (97%)	1.10	-0.02
		No	45 (2%)	7 (3%)		
	Concerned about future frequency	Yes	1997 (91%)	225 (94%)	1.76	0.03
		No	192 (9%)	15 (6%)		
	Concerned about future severity	Yes	2005 (91%)	227 (93%)	1.58	0.02
		No	198 (9%)	16 (7%)		

* *p* < 0.05

Chi-square test results suggested meaningful relationships between legal status and reasons for noncompliance with evacuation orders (Table 3). In all instances, those who identified as IFLLS were more likely to report reasons for noncompliance, when compared to non-IFLLS respondents. More specifically, 184 individuals identifying as IFLLS reported noncompliance due to previously being okay after ignoring an order, 181 reported insufficient funds, 170 reported an inability to miss work, 165 reported insufficient transportation, 177 reported not having a safe place to evacuate/travel to, 175 reported not feeling safe leaving their area of residence, 169 reported not being physically well enough to evacuate, 181 reported requiring medical care, 175 reported an inability to evacuate pets, and 176 reported an inability to evacuate livestock; if proportionately equal to the non-IFLLS population, these values were expected to be 94, 111, 70, 77, 101, 90, 79, 67, 106, and 67, respectively. Analysis revealed a positive and significant relationship between identifying as IFLLS and evacuation noncompliance because of being okay after previously ignoring orders (Χ^2^ (1, *n* = 2,989) = 160.21, *p* < 0.000), insufficient funds (Χ^2^ (1, *n* = 2,989) = 95.22, *p* < 0.000), inability to miss work (Χ^2^ (1, *n* = 2,989) = 224.22, *p* < 0.000), insufficient transportation (Χ^2^ (1, *n* = 2,989) = 165.68, *p* < 0.000), not having a safe place to evacuate/travel to (Χ^2^ (1, *n* = 2,989) = 114.89, *p* < 0.000), not feeling safe leaving one’s area of residence (Χ^2^ (1, *n* = 2,989) = 145.58, *p* < 0.000), not being physically well enough to evacuate (Χ^2^ (1, *n* = 2,989) = 168.86, *p* < 0.000), requiring regular medical care (Χ^2^ (1, *n* = 2,989) = 296.45, *p* < 0.000), inability to evacuate pets (Χ^2^ (1, *n* = 2,989) = 88.63, *p* < 0.000), and inability to evacuate livestock (Χ^2^ (1, *n* = 2,989) = 275.39, *p* < 0.000).

**Table 3:** Comparison of reasons reported for disobeying evacuation orders between those who identified as IFLLS and those who did not

Variable		IFLLS		Pearson X^2^	Cramér’s V
		No (%)	Yes (%)		
Was previously okay after ignoring order	Yes	725 (35%)	184 (77%)	*160.21	0.26
	No	1347 (65%)	54 (23%)		
Insufficient funds	Yes	954 (46%)	181 (67%)	*95.22	0.20
	No	1136 (54%)	46 (33%)		
Inability to miss work	Yes	581 (26%)	170 (75%)	*224.22	0.30
	No	1619 (74%)	58 (25%)		
Insufficient transportation	Yes	672 (30%)	165 (72%)	*165.68	0.26
	No	1580 (70%)	64 (28%)		
No safe place to evacuate/travel to	Yes	874 (41%)	177 (78%)	*114.89	0.22
	No	1268 (59%)	50 (22%)		
Does not feel safe leaving area of residence	Yes	711 (34%)	175 (74%)	*145.58	0.25
	No	1384 (66%)	61 (26%)		
Not physically well enough to evacuate	Yes	651 (29%)	169 (71%)	*168.86	0.26
	No	1563 (71%)	68 (29%)		
Requires regular medical care	Yes	524 (30%)	181 (76%)	*296.45	0.34
	No	1733 (70%)	57 (24%)		
Inability to evacuate pets	Yes	900 (41%)	175 (73%)	*88.63	0.19
	No	1277 (59%)	64 (27%)		
Inability to evacuate livestock	Yes	516 (23%)	176 (75%)	*275.39	0.34
	No	1680 (77%)	59 (25%)		

* *p* < 0.05

**Fig. 1:**
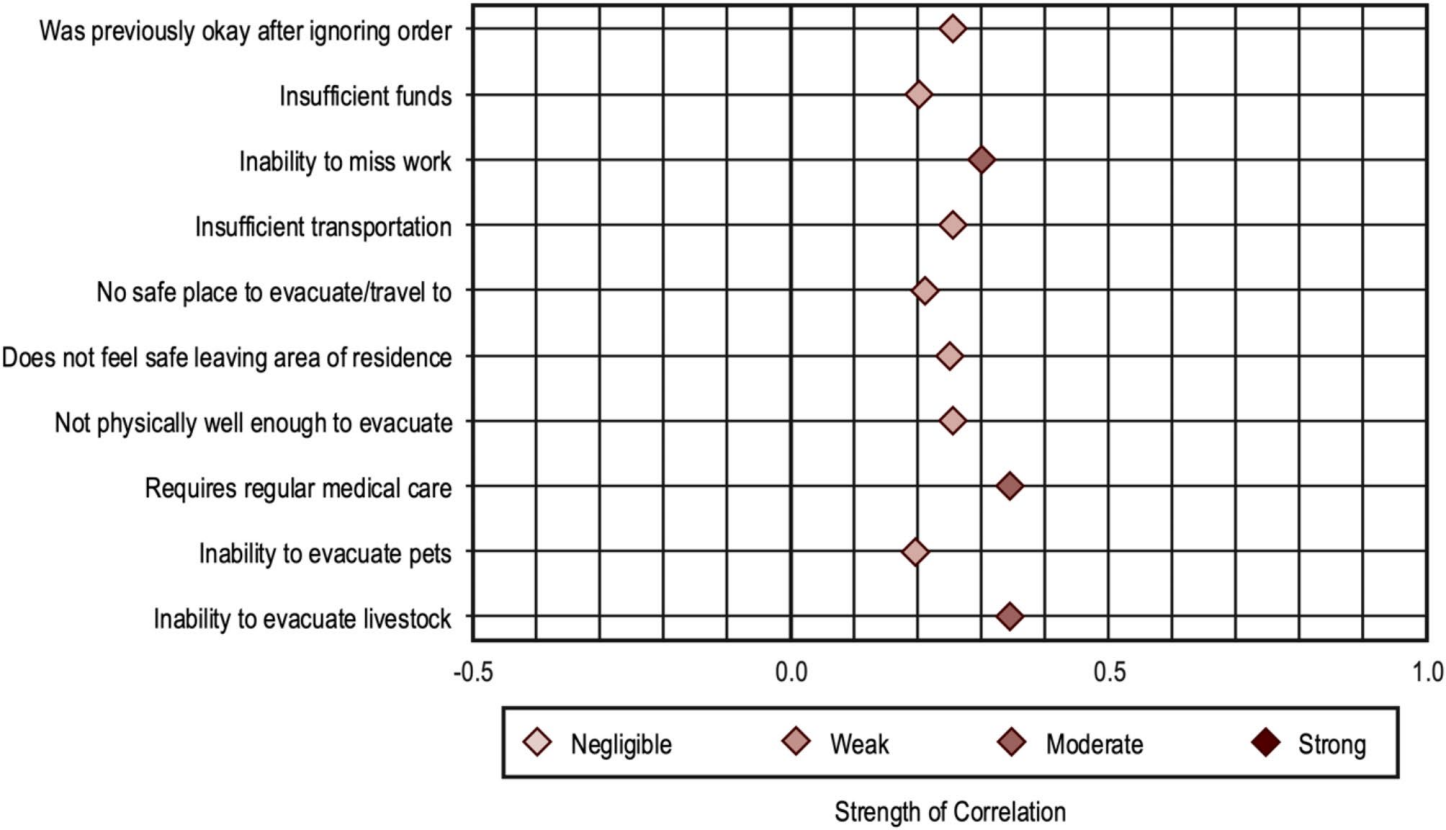
Distribution of Cramér’s V values: Correlations between legal status and reasons evacuation order noncompliance

Cramér’s V suggested weak, positive correlations between identifying as IFLLS and evacuation noncompliance because of being okay after previously ignoring orders (v = 0.26), insufficient funds (v = 0.20), insufficient transportation (v = 0.26), not having a safe place to evacuate/travel to (v = 0.22), not feeling safe leaving one’s area of residence (v = 0.25), not being physically well enough to evacuate (v = 0.26), inability to evacuate pets (v = 0.19); moderate, positive correlations existed between IFLLS and disobeying evacuation orders because of the inability to miss work (v = 0.30), requiring regular medical care (v = 0.34), and the inability to evacuate livestock (v = 0.34) (Fig. 1).

Noting the significant differences between those identifying as IFLLS in the US and those who reported they were non-IFLLS, we conducted additional sub-analyses to examine outcomes based on rurality (Table 4) and geographic location (Table 5) of the respondents.

**Table 4:** Comparison of reasons reported for disobeying evacuation orders between those who identified as IFLLS and those who did not by residential area

Variable	Residential Area														
	Urban					Suburban					Rural				
		IFLLS		X2	V		IFLLS		X^2^	V		IFLLS		X^2^	V
		No (%)	Yes (%)				No (%)	Yes (%)				No (%)	Yes (%)		
Was previously okay after ignoring order	Yes	222 (37%)	60 (77%)	*45.96	0.26	Yes	294 (32%)	67 (72%)	*59.04	0.24	Yes	209 (38%)	57 (85%)	*53.77	0.29
	No	382 (63%)	18 (23%)			No	625 (68%)	26 (28%)			No	340 (62%)	10 (15%)		
Insufficient funds	Yes	283 (47%)	61 (79%)	*28.48	0.20	Yes	375 (41%)	64 (75%)	*38.15	0.19	Yes	296 (52%)	56 (86%)	*26.67	0.21
	No	320 (53%)	16 (21%)			No	549 (59%)	21 (25%)			No	267 (48%)	9 (14%)		
Inability to miss work	Yes	176 (29%)	54 (71%)	*54.00	0.28	Yes	224 (23%)	65 (73%)	*106.47	0.31	Yes	181 (30%)	51 (81%)	*63.91	0.31
	No	434 (71%)	22 (29%)			No	770 (77%)	24 (27%)			No	415 (70%)	12 (19%)		
Insufficient transportation	Yes	236 (37%)	53 (67%)	*27.06	0.19	Yes	258 (26%)	61 (71%)	*78.29	0.27	Yes	178 (29%)	51 (80%)	*64.96	0.31
	No	407 (63%)	26 (33%)			No	746 (74%)	25 (29%)			No	427 (71%)	13 (20%)		
No safe place to evacuate/travel to	Yes	273 (45%)	52 (69%)	*15.77	0.15	Yes	345 (36%)	66 (80%)	*59.34	0.24	Yes	256 (44%)	59 (86%)	*43.42	0.26
	No	333 (55)	23 (31%)			No	604 (64%)	17 (20%)			No	331 (56%)	10 (14%)		
Does not feel safe leaving area of residence	Yes	203 (35%)	55 (69%)	*34.80	0.23	Yes	280 (30%)	64 (75%)	*71.83	0.26	Yes	228 (40%)	56 (79%)	*38.82	0.24
	No	385 (65%)	25 (31%)			No	656 (70%)	21 (25%)			No	343 (60%)	15 (21%)		
Not physically well enough to evacuate	Yes	200 (32%)	50 (62%)	*27.94	0.20	Yes	247 (25%)	66 (74%)	*95.22	0.30	Yes	204 (34%)	53 (80%)	*52.10	0.28
	No	427 (68%)	31 (38%)			No	738 (75%)	23 (26%)			No	398 (66%)	14 (20%)		
Requires regular medical care	Yes	170 (27%)	55 (71%)	*62.24	0.29	Yes	199 (20%)	71 (77%)	*149.52	0.37	Yes	155 (25%)	55 (81%)	*88.71	0.36
	No	469 (73%)	23 (29%)			No	806 (80%)	21 (23%)			No	458 (75%)	13 (19%)		
Inability to evacuate pets	Yes	258 (42%)	57 (70%)	*23.82	0.18	Yes	366 (38%)	65 (72%)	*40.60	0.19	Yes	276 (47%)	53 (78%)	*23.43	0.19
	No	361 (58%)	24 (30%)			No	604 (62%)	25 (28%)			No	312 (53%)	15 (22%)		
Inability to evacuate livestock	Yes	169 (27%)	59 (72%)	*68.06	0.31	Yes	194 (20%)	65 (74%)	*129.07	0.35	Yes	153 (26%)	52 (80%)	*77.93	0.35
	No	462 (73%)	23 (28%)			No	789 (80%)	23 (26%)			No	429 (74%)	13 (20%)		

* *p* < 0.05

**Table 5:** Comparison of reasons reported for disobeying evacuation orders between those who identified as IFLLS and those who did not

Variable	US Region																			
	Midwest					Northeast					South					West				
		IFLLS		X^2^	V		IFLLS		X^2^	V		IFLLS		X^2^	V		IFLLS			
		N (%)	Y (%)				N (%)	Y (%)				N (%)	Y (%)				N (%)	Y (%)	X^2^	V
Was previously okay after ignoring order	Yes	145 (38%)	30 (71%)	*16.89	0.20	Yes	114 (30%)	35 (80%)	*41.51	0.31	Yes	308 (38%)	86 (84%)	*80.07	0.29	Yes	158 (31%)	33 (66%)	*24.29	0.21
	No	232 (62%)	12 (29%)			No	261 (70%)	9 (20%)			No	507 (62%)	16 (16%)			No	347 (69%)	17 (34%)		
Insufficient funds	Yes	163 (45%)	30 (73%)	*11.91	0.17	Yes	171 (44%)	32 (74%)	*14.19	0.18	Yes	422 (51%)	81 (84%)	*38.85	0.20	Yes	198 (39%)	38 (81%)	*31.13	0.24
	No	201 (55%)	11 (27%)			No	216 (56%)	11 (26%)			No	407 (49%)	15 (16%)			No	312 (61%)	9 (19%)		
Inability to miss work	Yes	108 (29%)	30 (73%)	*32.82	0.28	Yes	96 (24%)	29 (67%)	*36.97	0.29	Yes	254 (30%)	77 (82%)	*101.03	0.33	Yes	123 (22%)	34 (68%)	*52.08	0.29
	No	267 (71%)	11 (27%)			No	309 (76%)	14 (33%)			No	598 (70%)	17 (18%)			No	445 (78%)	16 (32%)		
Insufficient transportation	Yes	140 (36%)	32 (73%)	*22.01	0.23	Yes	114 (28%)	30 (71%)	*33.85	0.27	Yes	278 (31%)	71 (80%)	*84.09	0.29	Yes	140 (25%)	32 (59%)	*28.23	0.21
	No	247 (64%)	12 (27%)			No	299 (72%)	12 (29%)			No	618 (69%)	18 (20%)			No	416 (75%)	22 (41%)		
No safe place to evacuate to	Yes	169 (45%)	31 (70%)	*9.97	0.15	Yes	158 (40%)	29 (73%)	*15.98	0.19	Yes	356 (42%)	82 (86%)	*66.33	0.27	Yes	191 (36%)	35 (73%)	*25.14	0.21
	No	204 (55%)	13 (30%)			No	240 (60%)	11 (27%)			No	485 (58%)	13 (14%)			No	339 (64%)	13 (27%)		
Does not feel safe leaving area of residence	Yes	130 (36%)	30 (67%)	*15.63	0.20	Yes	119 (31%)	33 (72%)	*30.48	0.26	Yes	303 (37%)	77 (81%)	*69.04	0.27	Yes	159 (30%)	35 (70%)	*31.83	0.23
	No	230 (64%)	15 (33%)			No	269 (69%)	13 (28%)			No	522 (63%)	18 (19%)			No	363 (70%)	15 (30%)		
Not physically well enough to evacuate	Yes	129 (34%)	29 (66%)	*17.82	0.20	Yes	114 (28%)	26 (63%)	*21.85	0.22	Yes	269 (31%)	77 (82%)	*95.50	0.31	Yes	139 (25%)	37 (64%)	*38.25	0.25
	No	256 (66%)	15 (34%)			No	294 (72%)	15 (36%)			No	599 (69%)	17 (18%)			No	414 (75%)	21 (36%)		
Requires regular medical care	Yes	106 (27%)	30 (70%)	*33.23	0.27	Yes	78 (19%)	31 (72%)	*61.87	0.36	Yes	234 (26%)	82 (85%)	*137.95	0.37	Yes	106 (19%)	38 (68%)	*67.86	0.33
	No	288 (73%)	13 (30%)			No	341 (81%)	12 (28%)			No	651 (74%)	14 (15%)			No	453 (81%)	18 (32%)		
Inability to evacuate pets	Yes	172 (46%)	30 (67%)	*7.05	0.13	Yes	177 (44%)	31 (72%)	*12.39	0.17	Yes	371 (43%)	76 (76%)	*38.62	0.20	Yes	180 (33%)	38 (75%)	*33.94	0.24
	No	204 (54%)	15 (33%)			No	226 (56%)	12 (28%)			No	487 (57%)	24 (24%)			No	360 (67%)	13 (25%)		
Inability to evacuate livestock	Yes	98 (26%)	32 (74%)	*43.29	0.32	Yes	77 (19%)	27 (66%)	*44.47	0.32	Yes	229 (27%)	82 (85%)	*136.92	0.38	Yes	112 (20%)	35 (64%)	*51.21	0.29
	No	284 (74%)	11 (26%)			No	321 (81%)	14 (34%)			No	635 (73%)	14 (15%)			No	440 (80%)	20 (36%)		

* *p* < 0.05

Respondents who reported identifying as IFLLS were more trusting of assistance compared to non-IFLLS. In this study, 218 of those who answered “yes” to the question about individual or family member legal status reported trusting assistance offered by neighbors, 211 reported trusting assistance offered by the local government, 205 reported trusting assistance offered by the National Guard, and 202 reported trusting assistance offered by the federal government; if proportionately equal to the non-IFLLS, these frequencies were expected to be 186, 193, 193, and 155, respectfully. This resulted in positive statistically significant relationships existed between IFLLS and trusting the assistance offered by neighbors during natural hazards (Χ^2^ (1, *n* = 2,989) = 26.73, *p* < 0.000), that offered by local governments (Χ^2^ (1, *n* = 2,989) = 10.48, *p* = 0.001), assistance offered by the National Guard (Χ^2^ (1, *n* = 2,989) = 5.82, *p* = 0.016), and assistance offered by the federal government (Χ^2^ (1, *n* = 2,989) = 29.41, *p* < 0.000). Cramér’s V suggested weak, positive correlations between IFLLS and trust in assistance offered by neighbors (v = 0.11) and the federal government (v = 0.11); negligible correlations existed between IFLLS and trust in assistance offered by local governments (0.07) and the National Guard (v = 0.05) (Table 6).

**Table 6:** Comparison of levels of trust in assistance offered by various entities during the response to natural hazards between those who identified as IFLLS and those who did not

Source of assistance		IFLLS		Pearson X^2^	Cramér’s V
		No (%)	Yes (%)		
Neighbors	Trust	1581 (76%)	218 (91%)	*26.73	0.11
	Distrust	495 (24%)	22 (9%)		
Local government	Trust	1719 (82%)	211 (90%)	*10.48	0.07
	Distrust	385 (18%)	23 (10%)		
National guard	Trust	1702 (85%)	205 (91%)	*5.82	0.05
	Distrust	307 (15%)	21 (9%)		
Federal government	Trust	1435 (72%)	202 (89%)	*29.41	0.11
	Distrust	549 (28%)	25 (11%)		

* *p* < 0.05

## Discussion

These findings suggest that individuals who identified as IFLLS are more likely to reside in areas that are prone to disasters. Moreover, they are more likely to have already experienced a disaster. These results support existing research that shows that marginalized groups such as immigrants without legal status and mixed-status households are disproportionately affected by disasters [4, 5]. Previous research also notes that disasters can widen existing disparities [20], meaning that not only are those who identified as IFLLS disproportionately affected, but that these disasters may contribute to disparities between these households and non-IFLLS households. While most of the literature related to the effect of natural hazards examines the ability of immigrants without legal status to understand and follow emergency warnings, the results from our study provide important information about the vulnerability of IFLLS to natural hazards based on where they reside. Understanding that IFLLS are at greater risk than those who are non-IFLLS can help shape federal, state, and local level emergency preparedness and response efforts. Specifically, policies and procedures should be developed that account for the unique needs of this population, their increased likelihood of being affected by natural hazards, and their concern about natural hazards, which are comparable to non-IFLLS.

One important element to note is that of the survey respondents who reported that they were IFLLS, the largest percentage identified as white. This runs contrary to the reported demographic makeup of immigrants without legal status in the US, who are primarily Hispanic or Latino [1]. And, while the number of undocumented individuals from former Soviet countries has increased since 2019, they remain a much smaller percentage of the undocumented immigrant population [1]. It is possible that IFLLS who are white felt more comfortable responding to the question about legal status since most negative rhetoric about those without legal status in the US is directed at Hispanic and Latino immigrants. Relatedly, the targeting of this ethnic group in the current political rhetoric may have suppressed response rates from these individuals.

The results from this study also show that respondents who identified as IFLLS are more likely than non-IFLLS to have an evacuation plan and emergency kit. However, results demonstrate that IFLLS face unique barriers that prevent them from complying with evacuation orders issued by government officials. For example, findings from this study suggest that IFLLS are less likely to follow evacuation orders because they have no safe place to travel to or do not feel safe leaving their area of residence. These results support those obtained by previous research efforts that found that immigrants without legal status often do not evacuate because they don’t know where to evacuate to [5]. The findings presented here, however, expand on the current research regarding barriers to disaster behaviors for IFLLS in the US by demonstrating that, when compared to non-IFLLS, these populations are more likely to not evacuate due to the cost of evacuating, an inability to miss work, not being physically well enough to travel, not having sufficient transportation, and requiring regular medical care.

Additionally, findings from this study add to the understanding of barriers to disaster evacuation and compliance with other disaster orders and directives for IFLLS because it is the first study to examine the impact of pets and livestock on evacuation compliance for this group. The findings from this study show that IFLLS are less likely to follow evacuation orders because they would have to leave their pets behind. The importance of including pets in evacuation plans was highlighted following the response to Hurricane Katrina. In 2006, the Pets Evacuation and Transportation Standards Act was passed, which required states to include pets in their evacuation and emergency shelter plans [21]. Despite the progress that has been made in reducing pets as a barrier to evacuation compliance, findings from this study suggest that IFLLS still experience pets as a barrier at a higher rate than non-IFLLS. This difference could be due to concerns about accessing official emergency shelters for fear of disclosing their legal status [10, 22] but understanding the specific reasons for experiencing barriers was outside the scope of this study.

Results from this study also show that IFLLS are more likely than non-IFLLS respondents to report that they would not comply with evacuation orders because of an inability to evacuate livestock. Evacuating livestock is logistically challenging and there is limited discussion of livestock evacuation plans [23]. The US Department of Agriculture provides guidelines for developing livestock evacuation plans, but to date, the responsibility for planning and executing livestock evacuation falls on livestock owners [24]. All livestock owners, regardless of IFLLS status, face the same challenges in livestock evacuation, but findings from this study suggest that IFLLS livestock owners may have diminished ability to move their livestock to safety and subsequently evacuate themselves from the disaster zone. Understanding the nuances of this difference is a topic for future research and has implications for improving disaster preparedness and response in agricultural communities.

Lastly, findings demonstrate, perhaps somewhat unexpectedly, that compared to non-IFLLS, respondents who identified as IFLLS have higher levels of trust in their neighbors, local governments, the National Guard, and the federal government. Most of the existing literature shows a lack of trust in government and negative mental health outcomes due to immigration policy and enforcement among immigrants without legal status [25–32]. While immigration policies have most frequently been found to increase fear, stigma, and discrimination, there is some evidence that policies such as the Deferred Action for Childhood Arrivals program reduce distress among those without legal status [33]. Additionally, a previous study found that, among Latinos, immigrants have the highest levels of trust in government and that non-immigrant Latinos tend to express similar levels of trust in government compared to Anglo demographic groups [34]. Additionally, some studies have found that immigrants in the US generally report a higher level of trust in government compared to native-born Americans [35, 36]. A recently published study by Tryberg also found that trust in political institutions among immigrants is particularly influenced by individual-level and contextual factors [36]. As applied to our results, this means that education level, time spent in the US, and previous exposure to democratic governance may have an outsized impact on trust levels among respondents who identified as IFLLS.

It is possible that social capital also plays a role in understanding the counterintuitive findings regarding trust and immigration status. Social Capital Theory argues that interpersonal trust serves as a building block for institutional trust, and that social networks are critical to the functioning of democracy [37–40]. Therefore, higher trust in neighbors, which is reflected in our findings, may directly spill over into higher levels of trust in political institutions. Put another way, if respondents who identified as IFLLS, also report having strong, positive social networks, based on the tenants of Social Capital Theory, this interpersonal trust could create greater institutional trust (i.e., trust in the National Guard, local, state, and federal governments).

Lastly, these findings may reflect the high number of US military veterans in the study population of respondents who identified as IFLLS. Previous research finds that veterans have higher voter turnout than non-veterans and are less cynical in their political attitudes [41, 42], suggesting that they may have higher levels of trust in political institutions. The high number of veterans in our sample of IFLLS respondents could also suggest that trust in disaster situations differs from trust in non-disaster situations, particularly among those with military experience. These findings require further study and exploration.

### Limitations

This study has several limitations. First, the design of the study serves as a limitation because distribution was done through a web-based survey tool. This inherently limited data collection to individuals who were literate, had access to a computer or cellphone, and an ability to access the internet. Additionally, this study was conducted using a cross-sectional survey. Therefore, the findings outlined are a one-time assessment of disaster preparedness and response in the US. All commonly cited limitations of cross-sectional surveys also apply to this study. Third, the survey was conducted using an online, non-probability sampling platform. This is common practice in modern survey research, and several methods—including sampling quotas and attention checks—were used to mitigate several of the concerns that arise with this study design. Fourth, the wording of the question regarding legal status makes it impossible to look only at participants who lack legal status themselves. The wording, “Do you or anyone in your immediate family lack legal status in the United States” was used in efforts to increase response rates to the question, but it is important to note that not every individual who responded “Yes” to this question lacks legal status themselves. It is possible, therefore, that respondents included in the IFLLS category had legal status themselves but responded “Yes” because an immediate family member lacks legal status. It is also important to note that scholars have raised concerns related to the ecological fallacy, in which inferences based on individual data are aggregated to a higher collective level. We acknowledge that our study, like many national studies, runs this risk with regards to disaster experience and legal status. However, the risk of ecological fallacy in our study is no greater than that of other national surveys. Finally, there is a possibility of response bias, as those without legal status may be less inclined to participate in the survey for a variety of reasons (e.g., limited access to technology, a lack of trust in national surveys, etc.). Despite this, a strength of this study is that it represents the first nationwide study of disaster preparedness and response among IFLLS in the US and expands the literature on this population.

## Conclusion

Given the size of the population of IFLLS in the US, the increasing political and practical importance of disaster preparedness and response, and the current political climate that has prioritized issues related to legal status, understanding the intersection of immigration and natural hazards has implications for the well-being of communities and individuals. These findings demonstrate that respondents who identified as IFLLS are at higher risk of experiencing a natural hazard, have more barriers to complying with evacuation orders, and have higher levels of trust in government entities. These findings can influence disaster preparedness and response policy development. For instance, disaster preparedness policies should account for the vulnerability and unique challenges faced by IFLLS while also considering and reducing the barriers to evacuation compliance. Lastly, these findings suggest that respondents who identified as IFLLS believe that they can rely on assistance offered by neighbors, local government, the National Guard, and the federal government during a disaster, which has implications for emergency warning and disaster planning and response efforts. This trust may be leveraged to facilitate evacuation compliance and disaster preparedness among IFLLS in the US.

## Data Availability

The datasets used and/or analyzed during the current study are available from the corresponding author upon reasonable request.

## Abbreviations

US: United States
IFLLS: Individuals or those with immediate family members lacking legal status
